# A Novel Method to Identify Pneumonia through Analyzing Chest Radiographs Employing a Multichannel Convolutional Neural Network

**DOI:** 10.3390/s20123482

**Published:** 2020-06-19

**Authors:** Abdullah-Al Nahid, Niloy Sikder, Anupam Kumar Bairagi, Md. Abdur Razzaque, Mehedi Masud, Abbas Z. Kouzani, M. A. Parvez Mahmud

**Affiliations:** 1 Electronics and Communication Engineering Discipline, Khulna University, Khulna 9208, Bangladesh; 2 Computer Science and Engineering Discipline, Khulna University, Khulna 9208, Bangladesh; niloysikder333@gmail.com (N.S.); anupam@khu.ac.kr (A.K.B.); 3 Department of Computer Science and Engineering, University of Dhaka, Dhaka 1000, Bangladesh; razzaque@du.ac.bd; 4 Department of Computer Science, Taif University, Taif 21944, Saudi Arabia; mmasud@tu.edu.sa; 5 School of Engineering, Deakin University, Geelong, VIC 3216, Australia; abbas.kouzani@deakin.edu.au (A.Z.K.); m.a.mahmud@deakin.edu.au (M.A.P.M.)

**Keywords:** pneumonia, chest radiograph, medical image processing, deep learning

## Abstract

Pneumonia is a virulent disease that causes the death of millions of people around the world. Every year it kills more children than malaria, AIDS, and measles combined and it accounts for approximately one in five child-deaths worldwide. The invention of antibiotics and vaccines in the past century has notably increased the survival rate of Pneumonia patients. Currently, the primary challenge is to detect the disease at an early stage and determine its type to initiate the appropriate treatment. Usually, a trained physician or a radiologist undertakes the task of diagnosing Pneumonia by examining the patient’s chest X-ray. However, the number of such trained individuals is nominal when compared to the 450 million people who get affected by Pneumonia every year. Fortunately, this challenge can be met by introducing modern computers and improved Machine Learning techniques in Pneumonia diagnosis. Researchers have been trying to develop a method to automatically detect Pneumonia using machines by analyzing and the symptoms of the disease and chest radiographic images of the patients for the past two decades. However, with the development of cogent Deep Learning algorithms, the formation of such an automatic system is very much within the realms of possibility. In this paper, a novel diagnostic method has been proposed while using Image Processing and Deep Learning techniques that are based on chest X-ray images to detect Pneumonia. The method has been tested on a widely used chest radiography dataset, and the obtained results indicate that the model is very much potent to be employed in an automatic Pneumonia diagnosis scheme.

## 1. Introduction

Pneumonia refers to the acute respiratory infection that affects the air sacs of a person’s lungs. When a person inhales, his/her lungs get filled with Oxygen-rich air; but, due to infections, tiny air sacs within the lungs (medically known as alveoli) may fill up with fluid and pus instead. This condition restricts the person’s ability to breathe properly—and that is when a person is said to have developed Pneumonia. Apart from difficulty in breathing, the symptoms of Pneumonia include coughing with mucus, sweating, fever, chest pain, exhaustion, fatigue, headaches, nausea, vomiting, and loss of appetite. Bacteria, Virus, and Fungi are all are capable of causing Pneumonia. Viral and bacterial Pneumonia are contagious and capable of propagating from person-to-person. Usually, a healthy person gets affected by Pneumonia when they inhale air that is contaminated by a patient of Pneumonia through sneeze or cough. Fungal Pneumonia is typically spread through the environment, not by a patient [1]. Pneumonia is classified based on how people acquire the disease. It can be Hospital-acquired Pneumonia (HAP), which is developed during the period a person stays in the hospital, Ventilator-associated Pneumonia (VAP), which is developed during a patient’s ventilation, Community-acquired Pneumonia (CAP), which is formed within a non-medical environment, or Aspiration Pneumonia, which occurs when a person inhales or intakes Pneumonia-causing bacteria with food, drinks, or water.

The treatment of Pneumonia depends on the type of Pneumonia the patient has developed, his/her age, and the severity of the infection. However, the most crucial part of the treatment is the diagnosis of Pneumonia, which helps a physician to know whether a person showing multiple symptoms has actually developed some form of Pneumonia or not. This task is more clinically challenging if the patient has developed a form of HAP or VAP, as the associated symptoms may require more than 48 h to reveal [2]. Among many methods to diagnose Pneumonia, the method that is based on analyzing chest X-rays is widely used. However, a number of credible alternatives are also available. For instance, blood tests can be carried out to confirm the presence of any infection and to try ascertain the organism, which is causing it. However, in many cases, accurate identification is not possible only from blood tests’ results. Another alternative test is called “Sputum test”, where a sample of mucus is collected from the deep cough of a patient, and analyzed to find out the reason for the infection. Another substitute can be the “Pulse oximetry” test, which helps doctors to measure the amount of oxygen present in the blood. It is usually done with the help of a sensor that is clipped or taped onto the patient’s finger. Alternatively, the doctors can perform a Bronchoscopy, which is a straightforward way of examining the bronchi using samples of tissue or fluid that were collected with the help of a flexible tube. This test is also useful for diagnosing the presence of lung problems other than Pneumonia in patients. Pleural fluid culture, chest Computed Tomography (CT) scan, and Arterial Blood Gas (ABG) test are some of the lesser-used methods in Pneumonia diagnosis. Regardless of the method, if not diagnosed in time and goes untreated, Pneumonia can cause several long-term complications and permanent damage to the patient’s respiratory system and other associated organs, including lung abscess, lung failure, and even blood poisoning (septicemia) [3]. People that belong to any age group can develop Pneumonia, but children under the age of five and elderly people who are over 65 are mostly affected by it [2,4]. Pneumonia is responsible for the death of over 800,000 children around the world every year, which amounts to almost 16 percent of all child-deaths [5], according to the World Health Organization (WHO). Most of the children who lose their life suffering from Pneumonia live in the South-Asian and Sub-Sahara region.

Among many forms of diagnosis, examining the chest X-ray is probably the most common for detecting Pneumonia. In this method, a physician or a pathologist determines the presence of any infiltration within the X-ray image of a potential patient who has exhibited multiple symptoms of the diseases. If any infection is detected in the lower respiratory tract, then the person is identified as a “positive”. Additionally, examining chest X-rays reveals important information related to the exact location and extent of the infection, pleural effusion cavitation, the lobes that are involved in the process, and necrotizing pneumonia [2]. Therefore, analyzing chest X-rays is a vital and imperative stage in the diagnosis of Pneumonia. Unfortunately, the number of experts who have attained this skill and can exercise it impeccably is not adequate, and most of these capable individuals are hired by acclaimed medical institutions and pathological centers. People who live in the developing areas and below the poverty line do not often receive proper diagnosis and timely treatment, and, consequently, bear the risk of developing complicated medical conditions. Training more individuals to undertake the task of diagnosing Pneumonia can be a viable solution to the problem. However, such training requires considerable time and funding, and the trainees are prone to commit mistakes, leading to misdiagnosis until they acquire sufficient experience.

Another solution to this problem has emerged because of the recent breakthroughs in the field of Artificial Intelligence (AI), Machine Learning (ML), and Digital Image Processing (DIP). Researchers can now use machines to develop Computer-Aided Diagnosis (CAD) techniques to diagnose Pneumonia by analyzing the X-ray images of the chest [6] due to the advancements in these domains. Among other ML techniques, supervised deep-learning (DL) algorithms offer an elegant way for working with any form of images. These algorithms perform convolution operations to extract characteristic traits, known as “features”, from the images containing diverse information; then use those features to determine whether the images belong to the same category (i.e., contain similar information), known as a “class”, or to different categories. Based on this simple yet effective principal, supervised learning techniques can analyze and differentiate among countless images containing various objects, shapes, colors, and patterns that belong to numerous classes. The eminence of ML techniques has been appreciated by the researchers working in diverse domains, including Computer Science, Mechanical Science, Material Science, Bio-medical Engineering, Construction and Civil Engineering, and even Business Studies. Apart from being used in detecting Pneumonia, ML and DIP techniques are rapidly used in various types of bio-signal classification, such as Electrocardiogram (ECG) [7], Electromyogram (EMG) [8], Electroencephalogram (EEG) [9], human activity recognition (HAR) [10], skin disease detection and classification [11], Diabetic Retinopathy (DR) [12], Alzheimer detection [13], cancer detection [14], and in many other areas to solve detection and classification problems.

The idea of exploiting involving computers to diagnose Pneumonia is certainly not new. Actually, it dates back to the year 1963, when Lodwick et al. described a concept of converting chest X-ray images into numerical features and described a CAD technique for determining lung cancer (LC) [15]. Article [16] outlines the very first application of CAD in chest radiography. CAD brought a new dimension in the interpretation of X-ray images, and this field was extensively researched throughout the 70s and 80s [17]. Even before the advancements of deep-learning methods, non-deep decision tree-based methods, which are not so ideal for working with high dimensional data like images, were used in chest radiography. For instance, in 1989, Hong and Unrik published an article where they used generalize and specialize (GS), an attribute-based ML algorithm, in order to distinguish between Pneumonia and Tuberculosis (TB) cases based on the revealed symptoms; and, showed that their proposed method outperforms AQ11 and ID3, which were two emerging ML techniques at that time [18]. Since the beginning of the 21st century, the capacity of the machines has increased exponentially each year. This rise of computational power has allowed researches to implement many time-consuming and memory-hungry DL methods in practice, which were previously considered to be too complex for the machines. Consequently, novel CAD methods with high computational complexity and massive raw data got the chance to be experimented with and come into practical use. In 2007, Coppini et al. designed a Neural-network-based diagnosis method for Chronic Obstructive Pulmonary Disease (COPD) patients, where they mathematically analyzed the posteroanterior and lateral radiographs of the subjects with and without emphysema [19].

In 2011, Karargyris et al. provided a segmentation-based method for screening Pneumonia and Tuberculosis while using chest X-rays [20]. In 2014, Ebrahimian et al. proposed a solution to the tricky problem of discriminating Pulmonary Tuberculosis (PTB) and Lobar Pneumonia (PNEU) [21], Noor et al. described a method for distinguishing among PNEU, PTB, and LC using chest radiographs, where they extracted features using wavelet texture measures and principal component analysis (PCA) [22], and Abiyev et al. used Convolutional Neural Networks (CNN) for predicting the presence of chest disease through analyzing chest radiographs [23]. In 2016, Khobragade et al. designed an automatic detection scheme to detect lung diseases—TB, LC, and Pneumonia—by segmenting the images of lungs from chest radiographs, and then using Artificial Neural Network (ANN) for classification [24]. In 2017, Wang et al. carried out extensive experiments in order to separate eight different diseases and medical conditions related to thorax based on the evidence present in chest X-rays using Deep CNN (DCNN) as the classification algorithm [25]. In 2018, Guan et al. designed a DL model, named Attention Guided CNN (AG-CNN), to distinguish the eight conditions mentioned in the previous study [26]. Additionally, in 2018, Singh et al. assessed the accuracy of algorithms in detecting the abnormalities on frontal chest X-rays and assessing the stability and change in their findings over serial radiographs [27]. In recent times, Chandra and Verma proposed a binary classification method based on a few statistical features that were extracted from chest X-ray images for Pneumonia detection [28], and Togaçar et al. proposed a feature learning model based on 300 selected deep mRMR features extracted from chest X-ray images and three different DL algorithms for the detection of Pneumonia [29].

In this study, a novel ML method has been proposed for Pneumonia detection while using a multi-channel CNN model. Features have been extracted from chest radiographic images using three different DIP techniques, and they were simultaneously put into a CNN model to provide the algorithm with diverse information on the properties related to Pneumonia. Our approach differs from the previous ones in terms of the type of features extracted, the order in which the images were processed, and the architecture of the DL model. Together, the employed DIP and CNN algorithms provide a powerful model for Pneumonia diagnosis, as explained in the Results and Discussion section. The proposed model is capable of detecting the presence of Pneumonia with a high degree of reliability; however, it is not optimized in order to determine the type of disease. In order to ensure easy interpretation, necessary graphs, charts, tables, and other illustrations have been included while describing the operating principle of the method.

The rest of the paper is organized, as follows. Section 2 explains the underlying principles of the employed methodology along with short discussions on its building blocks. Section 3 provides details on the experimental setup, presents the acquired results, and imparts brief discussions on the outcomes where necessary. Finally, Section 4 provides an overview of the work done in this article as well as indicates some room for future exploration in this area of research.

## 2. Methodology

**Figure 1:**
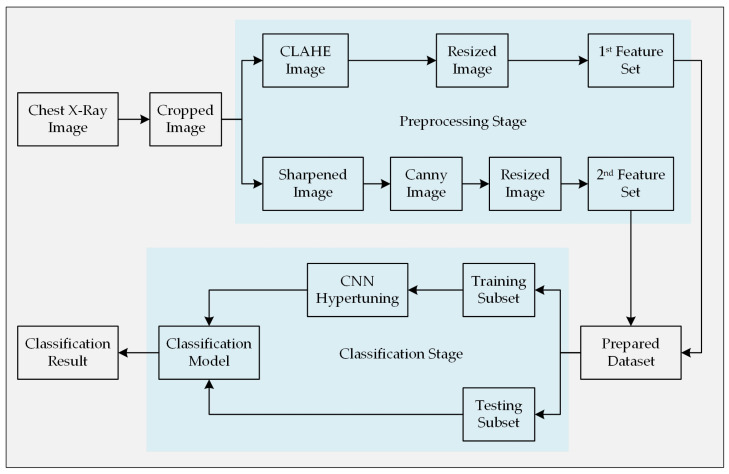
Methodology of the proposed automatic Pneumonia diagnosis model.

As stated earlier, this paper propounds an automatic Pneumonia detection algorithm though analyzing chest X-ray images and collecting features from them using a customized CNN architecture. A set of chest X-ray images containing both the Pneumonia-positive and Pneumonia-negative instances is required for training the supervised learning model. These sample images were collected from a widely used public dataset. First of all, the images were cropped from the center in order to omit unnecessary information outside of the chest-area and close to the border of the images. This step will help the algorithm to focus on the information relevant to this classification problem as well as to reduce the complexity of the algorithm. Subsequently, the corresponding images were preprocessed using two different image processing techniques to draw out potential features that are more relevant while distinguishing them. Two different techniques were implemented with the intention to bring diversity within the set of features that represents an image in the classification stage. Since all of the images of the employed dataset do not have the same dimension (height and width in terms of pixels), a resize operation was performed to ensure equal dimensionality. Figure 1 illustrates the entire methodology step-by-step. Finally, the acquired results of the experiment were presented, and the performance of the model was compared with other similar models.

### 2.1. Chest Radiograph Dataset

Images that were used to build this diagnostic model were collected from a renowned chest X-ray image repository [30], which contains a total of 5856 images. Among those images, 4274 samples were collected from the patients of Pneumonia, whereas the rest of the images were collected from healthy subjects who did not have Pneumonia. The chest X-rays were collected from pediatric patients that were aged between one and five years in Guangzhou Women and Children’s Medical Center, Guangzhou. The data collection procedure was performed alongside the patients’ routine clinical care. After collection, all of the images were passed through a grading system that was comprised of multiple layers. Each image was labeled based on the information that fits the corresponding patient’s most recent diagnostic profile. First of all, all of the unreadable and low-quality scans were marked and excluded. The remaining images were then labeled by two expert physicians to prepare them for ML applications. Lastly, a third expert checked the images one more time in order to suppress any error in labeling before including them in the dataset. The associated repository provided the images in three separate folders for training, validation, and testing purposes [30]. However, their contents were combined and re-separated based on their labels for the experiments that are described in this study.

**Figure 2:**
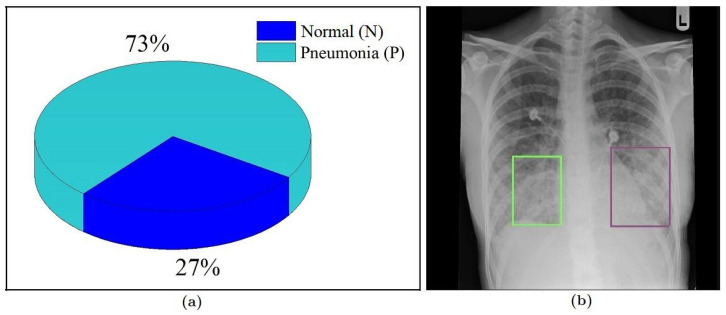
(**a**) Ratio of class samples in the dataset and (**b**) areas (marked) within the ribcage where typically indications of Pneumonia reside [31].

Figure 2a shows the proportion of the available images in the corresponding classes, which clearly indicates that the dataset is imbalanced in nature. The number of images collected from the Pneumonia-positive patients is roughly three times the number of samples that were collected from healthy people. Training a learning model using an imbalanced dataset often leads to decisions that are biased towards the superior class (i.e., the class with the larger sample size). This issue can be addressed by increasing the number of samples of the inferior class up to a point where the classes have an equal ratio (or close to that in terms of sample size) through using various image augmentation techniques. However, increasing the number of images to try to balance the dataset will significantly increase the overall size of the dataset and will not add any new information other than the original images. Therefore, in the experimental section of this study (discussed in Section 3), we took a different approach to address this problem. We ensured that the predefined train-test-split ratio (in our case 70–30) is maintained while splitting the images into the training subset and testing subset (i.e., the training subset would have 70% samples of both classes and the testing subset would have the remaining 30%). If the samples were divided without preserving this condition, most of the samples of the superior class (Pneumonia-positive) could have ended up in the training subset forcing most of the samples of the inferior class (Pneumonia-negative) to the testing subset. A split like this would not give the algorithm enough samples to analyze and properly “know” the later class, which might lead to high mis-classification rate of that class jeopardizing the model’s efficacy. This approach might not solve the problems that come with imbalanced data altogether, but it can reduce their effects in the classification stage.

### 2.2. Image Cropping

All of the areas within an image do not carry the same level of information pertinent to the objective. We want the learning program to analyze the regions of interest within the image (i.e., where the distinctive features are) related to the task and ignore or subside the other areas to reduce the complexity and execution time. In this case, we goal was to make the algorithm more focused around the center of the image to the lower section of the rib cage (as shown in Figure 2b). Since there is no information regarding the presence of Pneumonia outside the cage, a crop operation was performed on all of the X-ray images to omit most of the black regions near the left and right border before progressing them any further.

### 2.3. Image Preprocessing

Image preprocessing techniques are useful for improving the quality of an image or to reveal more relevant information on the targeted object. Image enhancement techniques fundamentally require applying a series of mathematical operations on the sample images. In X-ray imaging, the amount of photons absorbed by different types of tissues in the targeted region is taken into account [32]. Bones are very dense, which is why they absorb most of the photons coming from the X-ray beam. Other tissues, like skin and meat, absorb fewer photons and allow most of the ray to pass through them. This phenomena creates an image on the radiographic film that was placed opposite the X-ray beam and behind the subject with the variation of white, black, and gray colors. A physician then uses this image to observe the status of the internal organs. However, as a result of an abrupt variety of gray-level data, sometimes the information of the targeted area becomes misleading. In these cases, the properties of images need to be modified to obtain a better view of the targeted object. There are several image enhancement techniques available. In this work, we have utilized the Contrast-Limited Adaptive Histogram Equalization (CLAHE) method and an image sharpening technique to sharpen the images. Instead of collecting a single set of features from the input images, we extracted two different sets of features to provide the learning algorithm two different viewpoints on the training data. The architecture of the proposed CNN model is designed, such that the first channel processes the X-ray images whose contrast have been enhanced by the CLAHE method, and the second channel processes an edge-enhanced version of the input images along with a few intermediate preprocessing operations.

#### 2.3.1. Data Preparation for the 1st Channel

In the 1st Channel, the contrast of the image is enhanced by the CLAHE method [33,34]. In this method, the contrast of the input image is enhanced by utilizing its local histogram information. The objectives of this operation are to provide an upper bound on the amplification of histogram and to perform a clipping operation to keep the gray level information within a limit. Instead of using global histogram information, this technique operates based on the local histogram information of the image by dividing the entire image into small non-overlapping cells. The working procedure of the applied method has been depicted in the following algorithm:

Step 1: divide the whole image into R disjoint non-overlapping regions, where each region contains m×n pixels.
Step 2: calculate histogram ($H_{r}$) for each region r∈R.
Step 3: clip $H_{r}$ using a clipping threshold d where,
  (1) $$\begin{array}{c} \begin{array}{cc} d & =\frac{m\times n}{L}\left(1+P\right) \end{array} \end{array}$$
  and,
  (2) $$\begin{array}{c} \begin{array}{cc} P & =\frac{B}{100}\left(S_{max}-1\right). \end{array} \end{array}$$
  Here, L is the number of gray level, B∈[0,1] which is a clip factor, and $S_{max}$ is the maximum allowable slope.

Let, C(.) represent the CLAHE operator, which has been performed on $F^{\prime }\left(x,y\right)$ image. Then, the contrast enhanced image can be represented as

(3) $$\begin{array}{c} \begin{array}{cc} C(x,y) & =C\left(F^{\prime }\left(x,y\right)\right) \end{array} \end{array}$$

**Figure 3:**
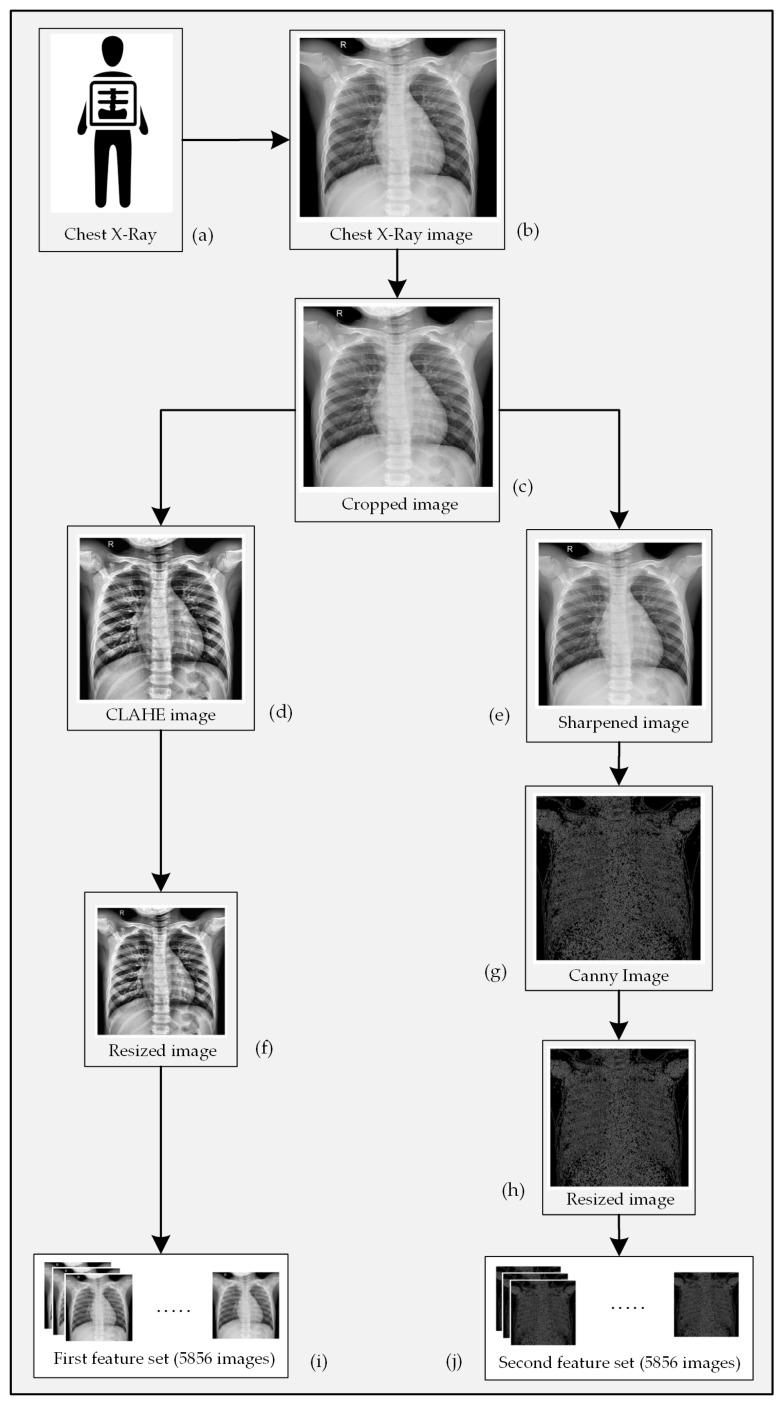
Processing of an X-ray image showing (**a**) chest radiograph collection, (**b**) conversion of 3(a) to a digital image, (**c**) elimination of unnecessary black pixels from 3(b), (**d**) CLAHE enhanced form of 3(c), (**e**) sharpened form of 3(c), (**f**) resized version of 3(d), (**g**) edge enhanced version of 3(e), (**h**) resized version of 3(g), and features collected from all X-ray images for the (**i**) first channel, and (**j**) second channel of CNN.

Figure 3d is a version of Figure 3c that has been enhanced through the CLAHE algorithm.

#### 2.3.2. Data Preparation for the 2nd Channel

An edge refers to a curve within an image along which the intensity of the image is changed rapidly typically due to encountering the boundary of an object. We have provided a set of edge enhanced image to the 2nd channel of our proposed CNN model derived from the original X-ray images. Edges within these images have been detected and enhanced using the Canny method. Prior to applying the method, an image sharpening operation was performed. This operation allows for the edges of the objects within an image to be more prominent. An image can be sharpened by subtracting a reasonable amount of blurred information from the original image while using the unsharp masking method [35]. A sample image $F^{\prime }\left(x,y\right)$ can be transformed to a sharpened image S(x,y) by applying the following algorithm:

Step 1: calculate the blurred image (B(x,y)) from the original image ($F^{\prime }\left(x,y\right)$) using the function D{}, such that
  (4) $$\begin{array}{c} B\left(x,y\right)\leftarrow D\{F^{\prime }\left(x,y\right)\} \end{array}$$
Step 2: subtract $F^{\prime }\left(x,y\right)$ from B(x,y) to obtain the edge enhanced image E(x,y), such that
  (5) $$\begin{array}{c} E\left(x,y\right)\leftarrow F^{\prime }\left(x,y\right)-B\left(x,y\right) \end{array}$$
Step 3: finally, acquire the sharpened image (S(x,y)) through the following operation
  (6) $$\begin{array}{c} S\left(x,y\right)\leftarrow F^{\prime }\left(x,y\right)-E\left(x,y\right) \end{array}$$

Figure 3e represents a sharpened version of the sample image that is depicted in Figure 3c. An edge represents the occurrence of an abrupt change of color in a specific area of an image. A CNN model performs kernel operations all-over an image to extract the global features. Images that only contain the edge information can be a valuable source of prominent features at the classification stage. There are quite a few edge detection techniques available. We have selected the Canny method to extract the edge information from the input X-ray images [36]. The Canny method performs the following operation to detect edges within an image:

- Perform a Gaussian filtering operation $G_{\sigma }\left\{\cdot \right\}$ through all over the images such that
  (7) $$\begin{array}{c} S_{b}\left(x,y\right)\leftarrow G_{\sigma }\left\{S^{\prime }\left(x,y\right)\right\} \end{array}$$
- Calculate the magnitude and angle of gradient m and a, such that
  (8) $$\begin{array}{c} m=\sqrt{{S_{bx}}^{2}+{S_{by}}^{2}} \end{array}$$
  (9) $$\begin{array}{c} a=\arctan\frac{S_{by}}{S_{bx}} \end{array}$$
  here $S_{bx}$ and $S_{by}$ represent the horizontal and vertical gradients, respectively.
- Calculate the threshold image $S_{t}\left(x,y\right)$ from $S_{g}\left(x,y\right)$, based on the threshold value δ.
- Perform a non-maximal suppression on the edges of image $C_{t}\left(x,y\right)$, such that
  (10) $$\begin{array}{c} S_{nm}\left(x,y\right)\leftarrow S_{t}\left(x,y\right) \end{array}$$
- Perform a hysteresis liking operation on image $S_{nm}\left(x,y\right)$ such that
  (11) $$\begin{array}{c} S_{h}\left(x,y\right)\leftarrow S_{nm}\left(x,y\right) \end{array}$$

Figure 3g is an edge enhanced version of Figure 3e derived while using the Canny method.

### 2.4. Pneumonia Identification Using Multichannel CNN

As discussed earlier, we used a CNN to extract global features from the X-ray images of the dataset, and used those features to detect the presence of Pneumonia in them. The CNN performs numerous convolution operations through-out the whole input image and, thus, extracts global features from the feature maps [37]. Let $x_{i}^{l}$ represent *i*th feature map at the *l*th layer, and $k_{i,j}\in R^{k_{1}\times k_{2}}$ represent the kernel matrix. Subsequently, the $j^{th}$ output map of layer l+1 can be represented as

(12) $$\begin{array}{c} x_{j}^{f,l+1}=F\left(u_{i}\right) \end{array}$$

where,

$$\begin{array}{c} u_{i}=\sum_{i\in o_{j}}x_{i}^{l}★k_{i,j}^{k_{1}\times k_{2}}+b_{j}^{l+1} \end{array}$$

here, $b_{j}^{l+1}$ is the bias value. An additional layer, called the sub-sampling layer, is implemented to reduce the overall computational complexity. Let S{.} represent sub-sampling function; then, the output of this sub-sampling layer can be represented as

(13) $$\begin{array}{c} x_{j}^{s,l+1}=S\left\{\beta^{l+1}\left(x_{j}^{f,l+1}+b_{j}^{l+1}\right)\right\} \end{array}$$

Whether it is a traditional Neural Network or a CNN, the last layer of the network is a Fully-Connected (FC) layer. Let the output of the last layer be represented as

(14) $$\begin{array}{c} O^{l}=L\left\{y^{last}w^{last-1}-b^{last}\right\}, \end{array}$$

here, L represents the logistic function and $O^{l}$ represents the corresponding output. The output of the network is different from the original output $q^{l}$ due to the random selection of the weight W and bias b values. The difference between the real output and the predicted output produces an error, which can be expressed as

(15) $$\begin{array}{c} E_{q}=\left|\right|O_{q}^{l}-t_{q}^{l}{\left|\right|}^{2} \end{array}$$

here, q∈{0,1} represents the number of classes. Currently, the total error can be calculated using

(16) $$\begin{array}{c} E=E_{0}+E_{1}. \end{array}$$

The minimum value of E provides the best output result, which can be calculated through the back-propagation error. This back-propagation ultimately provides the optimum values of (W,b). The values of (W,b) can be updated as

(17) $$\begin{array}{cc} W_{new} & =W_{old}-\beta \frac{\partial (E)}{\partial W}\\ b_{new} & =b_{old}-\beta \frac{\partial (E)}{\partial b} \end{array}$$

here, β is the incremental factor. Equation (17) is known as Stochastic Gradient Decent (SGD) method. However, this work has utilized the Adaptive Moment Estimation (ADAM) method for the optimization, such as

(18) $$\begin{array}{cc} W_{new} & =W_{old}-\beta \frac{{\hat{m}}_{t}}{\sqrt{{\hat{v}}_{t}}+\epsilon } \end{array}$$

(19) $${\hat{m}}_{t}=\frac{m_{t}}{1-\beta_{1}^{t}}\left\{\begin{array}{c} \mathrm{Bias}\;\mathrm{correction}\;\mathrm{for}\;\mathrm{the}\;\mathrm{first}\;\mathrm{moment}\;\mathrm{estimate} \end{array}\right.$$

(20) $${\hat{v}}_{t}=\frac{v_{t}}{1-\beta_{2}^{t}}\left\{\begin{array}{c} \mathrm{Bias}\;\mathrm{correction}\;\mathrm{for}\;\mathrm{the}\;\sec\mathrm{ond}\;\mathrm{moment}\;\mathrm{estimate} \end{array}\right.$$

(21) $$m_{t}=\beta_{1}m_{t-1}+\left(1-\beta_{1}\right)\frac{\partial (E)}{\partial W}\left\{\begin{array}{c} \mathrm{Updated}\;\mathrm{first}\;\mathrm{moment}\;\mathrm{estimate} \end{array}\right.$$

(22) $$v_{t}=\beta_{1}v_{t-1}+\left(1-\beta_{2}\right)\frac{\partial^{2}\left(E\right)}{\partial W^{2}}\left\{\begin{array}{c} \mathrm{Updated}\;\sec\mathrm{ond}\;\mathrm{moment}\;\mathrm{estimate} \end{array}\right.$$

**Figure 4:**
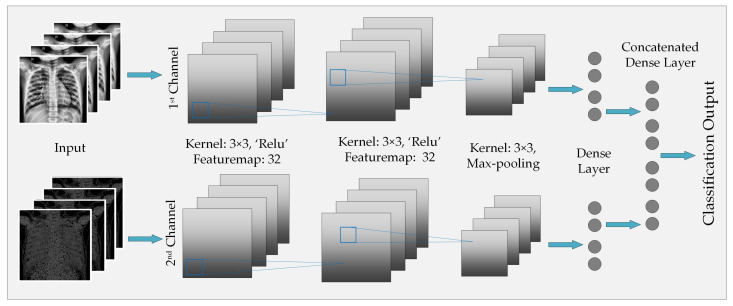
Architecture of the proposed multichannel CNN model for Pneumonia classification.

Figure 4 illustrates the architecture of the CNN model that has been implemented for global feature extraction and image classification. As the figure portrays, the 1st layer of the 1st channel contains seven feature maps, where we have employed a 2 × 2 kernel along with ReLU rectifier. 2nd convolution operation of the 1st channel also produces seven feature maps and it involves a 2 × 2 kernel. After the 2nd layer, another 2 × 2 kernel is utilized in order to perform a Max-pooling operation. After the Max-pooling operation, we can find the features extracted by the 1st channel at its Dense layer. The procedure is also the same for the 2nd channel. The only difference between the two channels is the type of image that they are given to process. After concatenating the two sets of features that were collected from the corresponding channels, a soft-max decision layer is employed to obtain the final decision regarding the presence of Pneumonia in the corresponding sample.

## 3. Results and Discussion

The solution to the problem in hand requires us to perform a binary image classification operation. As the mentioned dataset contains a set of X-ray images divided into two subsets based on the presence of Pneumonia in the corresponding patients, an experiment was designed to implement the CNN architecture that is illustrated and described in Section 2. The performance of the proposed method has been evaluated by different matrices, such as accuracy, precision, recall, and f-measure values. The model loss has been evaluated by two measuring parameters, namely, Binary Cross-entropy (BCE) and Kullback–Leibler (KL) Divergence values. Furthermore, Matthews Correlation Coefficient (MCC) values and Receiver Operating Characteristics (ROC) curve have also been provided to impart more depth to the model’s performance. Another factor that was taken into consideration is the dimension (height, width, and the number of channels) of the X-ray images tgar are present in the dataset. Theoretically, an image having a higher dimension contains more information than its lower-dimensioned counterparts and, in most cases, produces better classification results. However, increasing the dimensionality and resolution of images within a dataset also increases its overall size; and, processing large images requires more time and processing power than working with its smaller versions. Therefore, it becomes a matter of trade-off between performance (i.e., accuracy) and processing power as well as the required time while choosing the size of images in the dataset. In our experiment, we considered three different dimensions (height and width expressed in pixels) of the chest X-ray images— 64 × 64, 128 × 128, and 256 × 256, while keeping the information of all three color channels intact. The reason behind experimenting with three different image sizes is to find out the variation in the performance of our classifier with respect to image dimension and provide a rough estimation of the performance that can be acquired by implementing it in hardware of various capacities. Then again, these mentioned dimensions are not invariable; they can always be optimized to obtain the desired performance or lodge the algorithm to the available device by making necessary adjustments in the CNN architecture.

**Figure 5:**
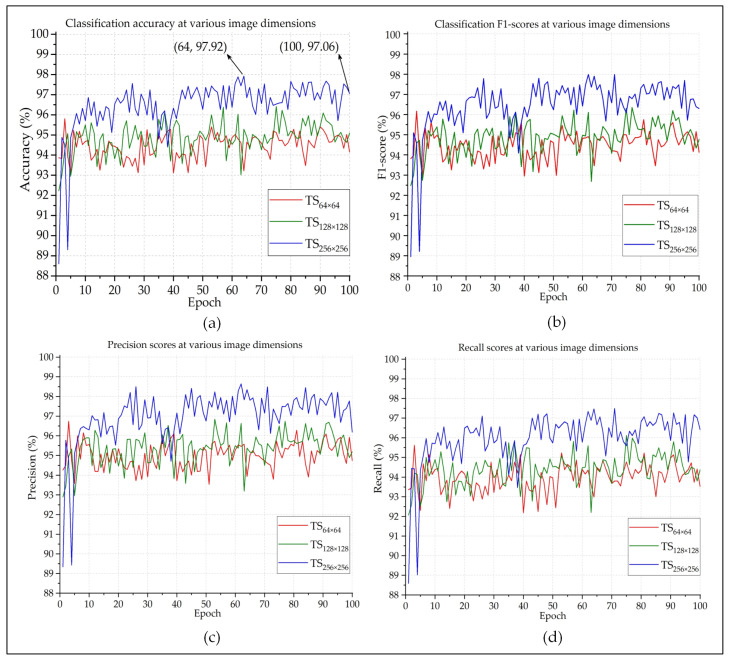
The (**a**) accuracy, (**b**) f-measure, (**c**) precision, and (**d**) recall of the performed classification.

Figure 5 presents the accuracy, precision, recall, and F1-score of the proposed method on the test samples (TS) for three different image sizes (denoted as TS_64 × 64_, TS_128 × 128_, and TS_256 × 256_). The experiment was carried out for 100 epochs. As expected, this graph shows that images with the 256×256×3 dimension provide the most amount of information and, hence, achieve the overall best performance when compared to the other two image sizes. According to Figure 5a, the recorded accuracy was 97.06% at the 100th epoch. However, the best classification accuracy was achieved at the 64th epoch, which is close to 98%. Figure 5c,d represent the precision and recall scores of each classification, respectively. Both of them are very common and widely used parameters when it comes to judging the performance of a classifier. Precision refers to the proportion of positive identifications that are actually correct; whereas, recall indicates the proportion of the actual positive instances that were accurately identified [38]. Although both of the parameters are equally important, they cannot individually provide an accurate estimation of the efficiency of the classifier.

**Figure 6:**
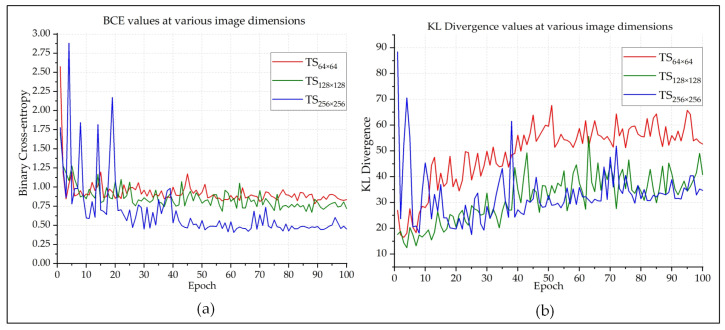
The (**a**) BCE and (**b**) KLD curves of the Pneumonia classifications.

**Figure 7:**
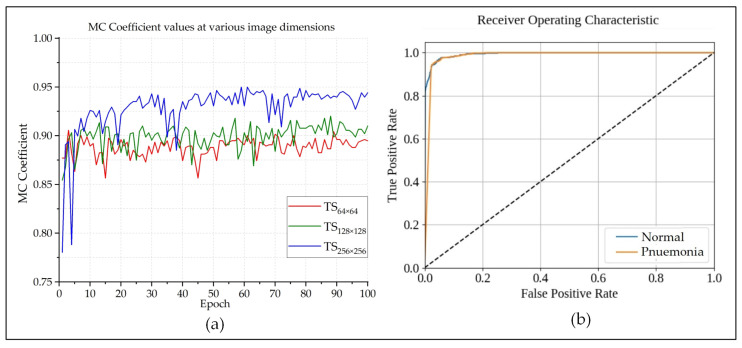
The (**a**) Matthews Correlation Coefficient (MCC) and (**b**) Receiver Operating Characteristics (ROC) curve of the performed classification.

F1-score (also known as F-measure or F-score) is a much more reliable parameter than precision and recall, since it calculates the harmonic mean of those two parameters. So much so, many experts believe that F1-score is a better arbitrator than the classification accuracy while quantifying the cogency of a classification algorithm. Figure 5b shows that the F1-score of each epoch is very close to the classification accuracy of that epoch. This indicates that the model is providing authentic outcomes, and it is not biased towards any class. Figure 6 presents the BCE and KL Divergences of the classifications. The BCE of a model should be as low as possible, and it would be 0 for a perfect classification outcome. Figure 6a shows that, as the model got better trained after each epoch, BCE continuously decreased. Because the larger images provided better classification outcome than the smaller ones, quite expectedly, their BCE curve is better than the others. The same also goes for the KL Divergences, which is depicted in Figure 6b. The MCC is another useful parameter while evaluating binary classifiers. The MCC values lie in between [−1,1]. An MCC value of −1 represents a binary classifier with the worst performance, and a value of +1 indicates a classifier with the best performance. The best MCC values achieved by the designed classifier was +0.95, according to Figure 7a. The ROC curve helps us to visualize the performance as a whole. Figure 7b presents the ROC curve of this experiment. The *X*-axis of this curve represents the False Positive Rate, and the *Y*-axis of this curve represents the True Positive Rate. The topmost left corner of the graph that is close to the (0,1) position, which means that the model classified most of the positive and negative instances correctly into their corresponding classes [39]. Table 1 provides an overview of the classification performances at different image dimensions in terms of the performance evaluating parameters described above. As discussed earlier, the results acquired while working with the 256×256×3 images are superior to those of the other two.

**Figure 8:**
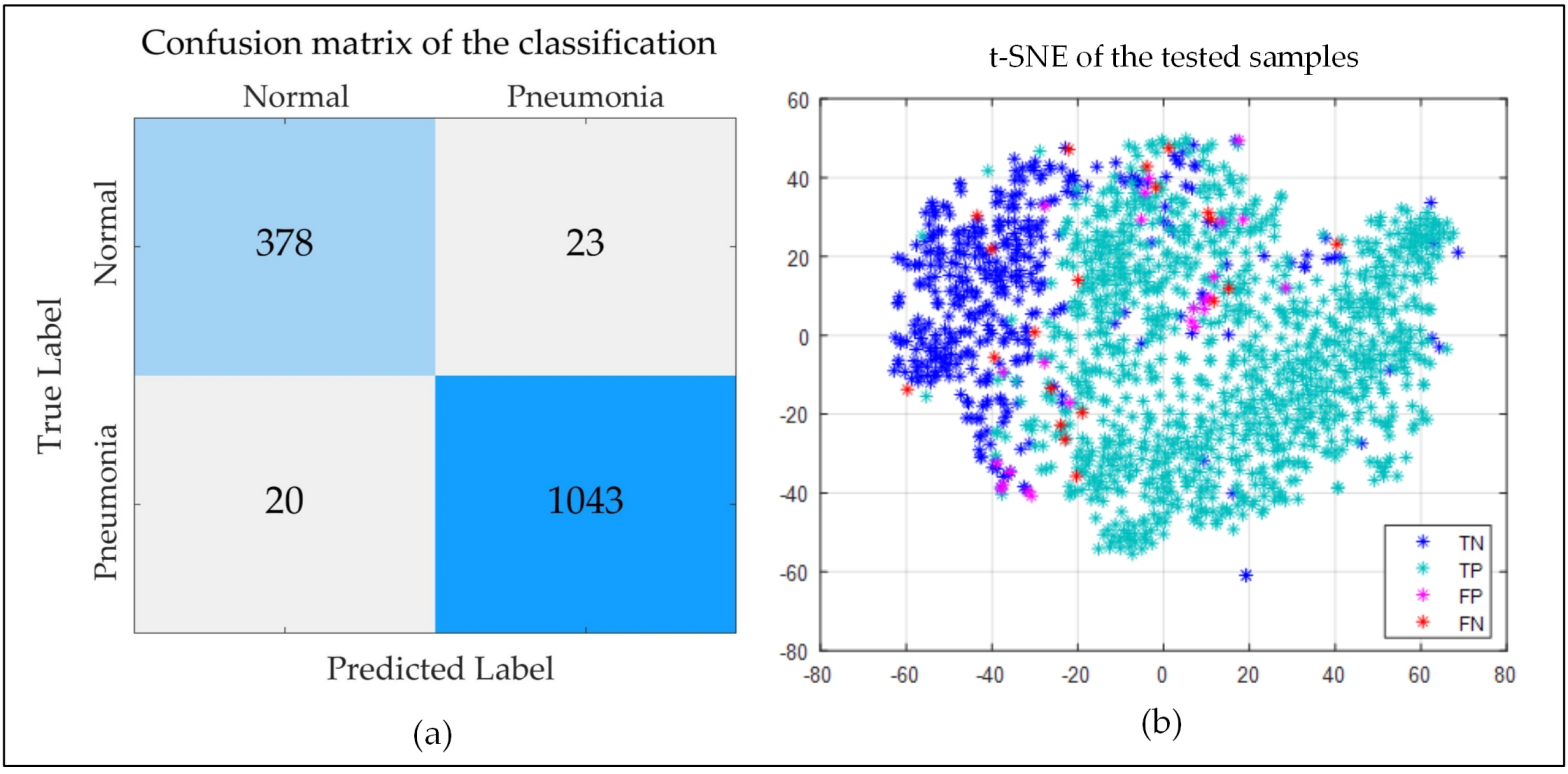
The (**a**) Confusion Matrix, and (**b**) t-SNE distribution of the tested samples.

Figure 8a demonstrates the confusion matrix of the last classification outcome (image dimension: 256×256×3, epoch: 100). It shows that only 43 samples among the 1464 testing samples were mis-classified by the described algorithm. Apart from them, 98.19% Pneumonia-positive cases and 94.26% healthy cases were correctly identified. Figure 8b shows the t-distributed Stochastic Neighbor Embedding (t-SNE) graph of the samples of the testing subset. Most of the Pneumonia-positive (true-positive, TP) samples are clustered at the right side of the graph, and most of the Pneumonia-negative (true-negative, TN) instances are clustered at the left, as we can see from the figure. However, there are a few areas where they overlap. False-positive (FP) and false-negative (FN) samples are scattered across the tw-dimensional (2-D) plane. This representation can be useful while hyper-tuning the model for better performance.

Table 2 presents a comparison of the acquired results of the proposed model with some of the state-of-the-art CNN-based Pneumonia diagnosis techniques. Abiyev RH [23] performed a CNN-based classification on the National Institute Health Clinical Center dataset. This dataset (known as ChestX-ray8 ) contains 112,120 frontal chest images [25]. The method described in [23] obtained a 92.40% classification accuracy. The authors of the study [40] reported an accuracy of 93.63% and an F1-score of 92.70% on the same dataset in the following year. The study described in [41] used transfer learning on the dataset referenced in [42] and, after performing a four-class classification, obtained an accuracy of 92.80%. The outcomes of the studies described in [43,44,45] are directly comparable to this work, since they all perform binary classifications and employ the same dataset. As the table shows, our method outperforms them by 4.19%, 1.56%, and 1.53%, respectively, in terms of classification accuracy. Although all of the cited articles did not mention all of the matrices necessary for a direct comparison (precision, recall, and F1-score), our method attained better scores than the ones that reported such information. Only the method described in [45] recorded a better recall score than the proposed model; however, it attained a lower F1-score due to poor precision. Based on these facts, it can be concluded that our method is superior in detecting Pneumonia from chest radiographs to other CNN-based methods.

## 4. Conclusions

This paper described a novel ML method to diagnose Pneumonia while employing a two-channel CNN. The acquired results suggest that the method is highly accurate in detecting whether a person has or does not have Pneumonia based on the radiographic image of their chest. The described method can provide better detection outcome than the contemporary ones, as it incorporates two different sets of features extracted from each training image. Additionally, the results of the conducted experiment suggest that increasing the size of the image (height and width in pixels) can potentially lead to a better classification outcome, as a large image usually contain more information than a smaller one. However, choosing to work with larger images significantly increases the size of the dataset, which in turn makes the model more complex, and it requires more time to build as well as a machine with high capacity to accommodate it. There will be a trade-off between the desired performance and collectible cost while designing the associated hardware to implement this model. We are looking forward to hyper-tuning the model more sophistically in order to achieve even better classification performance before implementing it in a device for online and automatic Pneumonia detection, which is the ultimate goal of this research project.

## Figures and Tables

**Table 1:** Summary of the classification outcome at the 64th epoch.

Image Dimension	Annotation	Accuracy%	Precision%	Recall%	BCE	KLD	MCC	F1-Score%
64 × 64 × 3	TS_64 × 64_	93.22	93.94	92.99	0.99	56.68	00.87	93.46
128 × 128 × 3	TS_128 × 128_	95.28	95.37	94.78	0.72	37.83	00.91	95.07
256 × 256 × 3	TS_256 × 256_	97.92	98.38	97.47	0.46	29.77	00.94	97.91

**Table 2:** Comparison of the acquired results.

References	Dataset	Class	Model	Accuracy (%)	Precision (%)	Recall (%)	F1-Score (%)
[23]	[25]	12	CNN	92.40	−−	−−	−−
[40]	[25]	2	CNN	93.63	93.9	93.00	92.70
[41]	[42]	4	CNN	92.80	87.2	93.20	90.10
[43]	[30]	2	CNN	93.73	−−	−−	−−
[44]	[30]	2	CNN	96.36	−−	−−	−−
[45]	[30]	2	CNN	96.39	93.28	99.62	96.35
This Work	[30]	2	CNN	97.92	98.38	97.47	97.97

“−−” denotes that the information is not mentioned in the associated paper.
